# The effect of epidermal growth factor on the pseudo-healing of traumatic tympanic membrane perforations

**DOI:** 10.1016/j.bjorl.2019.06.011

**Published:** 2019-07-27

**Authors:** Zhengcai Lou

**Affiliations:** Yiwu Central Hospital, Department of Otorhinolaryngology, Yiwu, China

**Keywords:** Wounds and injuries, Tympanic membrane rupture, Epidermal growth factor, Wound healing

## Abstract

**Introduction:**

Traumatic tympanic membrane perforations tend to heal spontaneously. However, in this study, several perforations exhibited abnormal healing, where the morphology of healing tympanic membranes differed from that of non-perforated tympanic membranes. Pseudo-healing of the tympanic membrane was characterized by the accumulation of thickened tissue in the perforated area.

**Objective:**

The purpose of this study was to evaluate the utility of epidermal growth factor in cases showing pseudo-healing of traumatic tympanic membrane perforations.

**Methods:**

A total of 26 traumatic tympanic membrane perforations showing pseudo-healing were included in this study. In all cases, tissue that accumulated in the perforated area was removed, which subsequently caused a new perforation to form. An epidermal growth factor solution was applied to the tympanic membrane once daily to keep the tympanic membrane moist. Closure rates and times were evaluated at 6 months.

**Results:**

During the 6 months follow-up period, two patients were lost. Of the remaining 24 patients, the closure rate was 100% (24/24) and the closure time was 6.1 ± 2.3 days (range: 3–12 days). The morphology of the healed tympanic membrane was not significantly different from that of the remnant tympanic membrane.

**Conclusions:**

Pseudo-healing of traumatic tympanic membrane perforations affects sound conduction. This can be associated with various symptoms, including tinnitus, aural fullness, and ear discomfort. The excision of excessive epithelial tissue and topical application of epidermal growth factor can correct the pseudo-healing of traumatic tympanic membrane perforations.

## Introduction

**Figure 1:**
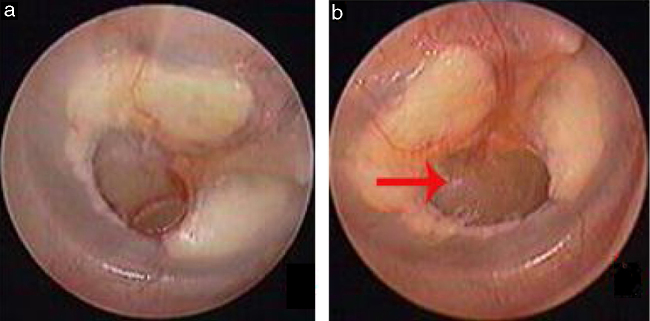
The spontaneous healing of traumatic TMP: 3 days after perforation (a), atrophic healing (b). Red arrows indicated atrophic eardrum.

**Figure 2:**
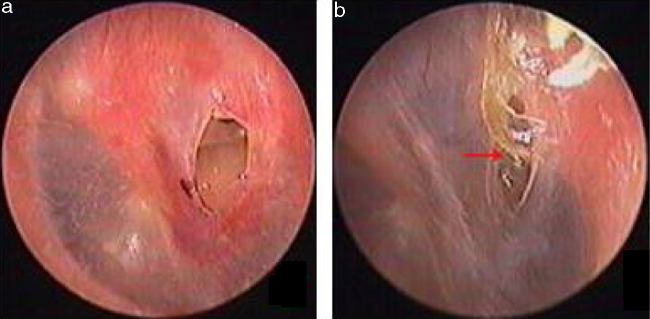
The spontaneous healing of traumatic TMP: 3 days after perforation (a), crust healing (b). Red arrows indicated crust.

**Figure 3:**
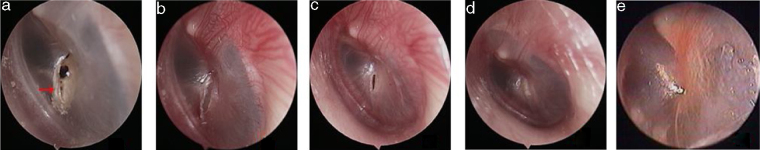
The pseudo healing of traumatic TMP and EGF treatment: the pseudo healing of eardrum within 5 months after perforation (a), a perforation was seen after thickened tawny tissue was removed (b), 4 days after EGF treatment (c), 10 days after EGF treatment (d), 2 months after EGF treatment (e). Red arrows indicated that thickened tawny tissue closed the perforation.

Traumatic Tympanic Membrane Perforations (TMPs) are commonly observed in otology clinics and typically have a high spontaneous healing rate. However, approximately 6%–20% of TMPs fail to heal properly, where persistent perforations result from abnormal healing^1^, ^2^, ^3^ which previously included atrophic and crust healing. Absence or weakness of the fibrous layer (i.e., when only an epithelial layer is present) in a healed tympanic membrane is characterized as atrophic healing (Fig. 1) and perforations covered by a thin and fractured crust are considered to denote crust healing (Fig. 2).^4^, ^5^ Although the perforation was closed in some cases in the clinic, the morphology of the healing tympanic membrane in the perforated area was different from that of the normal tympanic membrane.^3^ As the tympanic membrane healed, it exhibited thickened tawny tissue not representative of normal tympanic membrane morphology. A boundary line was evident between the healing and remnant tympanic membrane, and the thickened tissue (the color is tawny: it is called as tawny tissue for convenience in this paper) appeared to extend into the perforation, which we can define as pseudo-healing of the tympanic membrane (Fig. 3). The clinical features and treatment approaches for traumatic TMP pseudo-healing have not been well characterized, due to the limited number of cases and short follow-up periods of previous clinical studies.

Pseudo-healing increases the weight of the tympanic membrane because of the thickened tissue, thereby affects tympanic membrane movement and sound conduction, resulting in the symptoms of tinnitus, aural fullness and, in some cases, mild conductive hearing loss. In addition, perforations still remain when thickened tissue is removed from patients with pseudo tympanic membrane healing. Excision of the perforated edge, combined with patch treatment and myringoplasty, was previously performed to address any remaining perforations.^1^, ^2^ However, myringoplasty is a costly procedure and causes side effects.^1^, ^2^

Growth factors were used to repair chronic TMPs in recent years. Kanemaru et al.^6^ suggested that basic Fibroblast Growth Factor (b-FGF) enabled the regeneration of the chronic TMP without conventional operative procedures. Epidermal Growth Factor (EGF), a 53 amino acid mitogenic polypeptide present in many mammalian species, is one of several growth factors being investigated for its potential to expedite the healing process.^7^ Recent experimental and clinical studies have demonstrated that EGF improves the closure rate of human traumatic TMPs and experimental chronic TMPs, with no harmful effects on the middle or inner ear.^8^, ^9^, ^10^, ^11^ However, Ramsay et al.^12^ found that EGF was not effective for human chronic TMPs. This study describes the clinical features of tympanic membrane pseudo-healing and evaluated the effects of EGF on traumatic TMPs showing pseudo-healing.

## Methods

The present study was approved by the Institutional Ethical Review Board of Yiwu Central Hospital (nº 20091206). Informed consent was obtained from all participants.

Study subjects were recruited from among consecutive patients diagnosed with a traumatic TMP who visited the Department of Otorhinolaryngology, Head and Neck Surgery, at the Yiwu Central Hospital between January 2010 and December 2016. The inclusion criteria were: (1) traumatic TMP(s) for more than 3 months and (2) no evidence of perforation but the presence of thickened tawny tissue in the healing tympanic membrane, lacking the morphological appearance of the normal tympanic membrane. Age, gender, date and cause of the traumatic injury, TMP size, and the presence or absence of otorrhea were recorded at the time of the visit. All patients were examined with an endoscope after cleaning cerumen or blood clots from the External Auditory Canal (EAC) using a cotton bud soaked in povidone-iodine solution, and the site and size of the perforation were documented. The criteria for pseudo-healing of the tympanic membrane included: a perforated area covered by thickened tawny tissue, the absence of normal tympanic membrane morphology, a clear boundary line between the healing and remnant tympanic membrane, and observation of a perforation on excision of the thickened tawny tissue (Fig. 3). An EGF solution was applied to all patients during treatment.

### Technical method

The EAC was cleaned with a cotton bud soaked in povidone-iodine solution. Cotton wool pledgets moistened in 4% (w/v) lidocaine was placed in the surface of tympanic membrane three times for 5 min each time, then thickened tawny tissue was removed using micro-forceps by endoscopy, but the perforated edge was not further removed. No scaffolding material was used. Approximately 0.1–0.15 mL (or 2–3 drops) of recombinant bovine EGF solution (21,000 IU/5 mL; Yi Sheng, Zhuhai City, Guangdong Provice, China) was subsequently applied to the tympanic membrane along the EAC. EGF drops was self-administered by the patient once daily to keep the tympanic membrane moist, and the patients were advised to protect the ears against water.

All patients were followed-up twice a week until the perforation had closed completely or until 6 months post-treatment. EGF drops were self-applied by the patients at home as instructed, until complete perforation closure was confirmed by a physician. The physician also determined whether EGF self-application by patients was appropriate and confirmed if purulent otorrhea had developed within 2–3 days following commencement of the treatment. The eardrop dose was adjusted as needed to keep the tympanic membrane moist (i.e., not excessively wet or dry). Patients were advised to discontinue eardrops if their symptoms disappeared. Oral amoxicillin and ofloxacin otic drops were administered simultaneously if purulent otorrhea developed. To reduce clinician bias, tympanic membrane closure and development of otorrhea were photo-documented (via color photographs) during each follow-up visit.

### Post-treatment evaluation

All patients underwent a pure-tone test and tympanometry at the initial and final visits, or 6 months following their treatment. Pure-tone audiograms were obtained in a quiet room using an audiometer (Genemed Synthesis, Inc., San Antonio, TX, USA), including determination of the pure tone air and bone thresholds. Average ABGs were calculated at 0.5, 1, 2 and 4 kHz. The data were statistically analyzed with the paired *t* test (comparison of means). ***χ***^2^ test was used for categorical data of tympanogram. A *p*-value of <0.05 was considered to indicate statistical significance.

## Results

**Figure 4:**
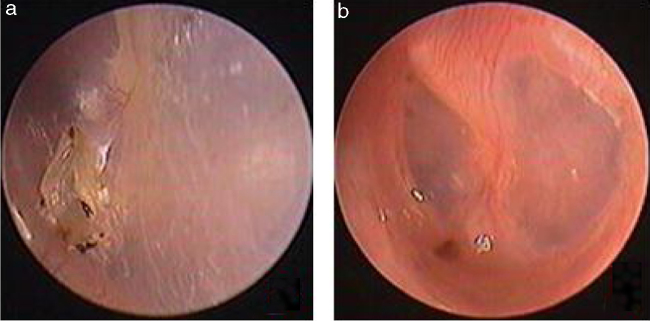
The pseudo healing of traumatic TMP and EGF treatment: the pseudo healing of eardrum within 2 months after perforation, thickened tawny tissue and a punctate perforation were seen (a); one week after EGF treatment (b).

**Figure 5:**
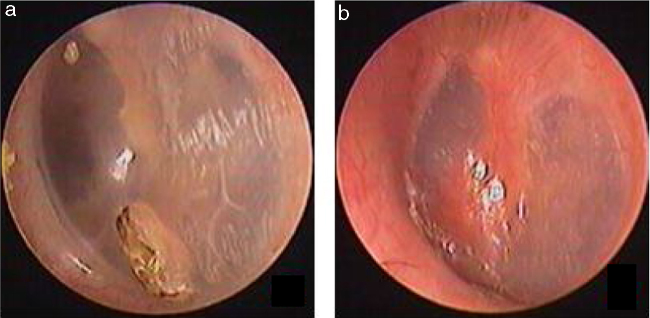
The pseudo healing of traumatic TMP and EGF treatment: the pseudo healing of eardrum within 2 months after perforation, thickened tawny tissue and a punctate perforation were seen (a); 10 days after EGF treatment (b).

In total, 26 patients with a mean age of 34.6 ± 12.8 years (range: 22–61 years) were treated. Of the 26 patients, 9 were male and 17 were female, and the mean treatment duration was 7.78 ± 1.48 months (range: 5–11 months). Of the 26 patients, 25 perforations were caused by a slap or blow to the ear while one perforation in resulted from a basketball injury. No otorrhea was observed in 26 patients. Four of the 26 patients had a perforated edge enclosed by thickened tawny tissue, as well as a crevice perforation, on endoscopy (Figure 4, Figure 5). In the remaining 22 patients, thickened tawny tissue covered the perforated area such that no perforation was visible. Perforation was observed when thickened tawny tissue was excised, and no bleeding or congestion occurred at the perforation edge. Medium-sized perforations (1/8–1/4 of the tympanic membrane) were found in 15 patients, and small-sized perforations (less than 1/8 of the tympanic membrane) were found in 11 patients. The pure-tone test demonstrated that the bone conduction threshold was normal in 26 patients. The air conduction threshold was measured at 0–10 dB in 23 patients and 10–20 dB in 3 patients. Tympanometry classified 19 patients as A-Type, as type C in 2 patients and 5 patients as B-Type before the thickened tissue was removed from the tympanic membrane.

Two patients were lost during the 6 month follow-up, and therefore were excluded. The final analysis only included the 24 patients in this study. Of the remaining 24 perforations, the patients did not develop the purulent otorrhea of the middle ear, the closure rate was 100% (24/24), and the mean closure time was 6.1 ± 2.3 days (range: 3–12 days). The symptoms before and after the treatment showed in the Table 1, of the 24 patients, tinnitus did not resolve in one patient while these symptoms at first visit resolved in 23 patients. The morphology of the healing tympanic membrane did not differ from that of the normal tympanic membrane (Fig. 3). All the patients only needed one bottle of EGF drops, the medical cost of which is 25 RMB (approximately 3.7$). In addition, the mean ABG improvement rate from 11.3 ± 4.5 dB at the initial visit to 9.8 ± 3.3 dB at 6 months post treatment was not statistically significant (*p* > 0.05); however, tympanometry indicated that all of the 24 patients were A-Type at 6 months post-treatment. No severe EGF-related complications, such as pain, severe vertigo, infection, or hyperplasia of the EAC, were observed during the treatment process.

**Table 1:** The symptoms before and after the treatment in 24 patients.

Symptoms	Prior treatment n (%)	Post-treatment, n (%)
Ear discomfort	22 (91.7%)	0 (0.0%)
Aural fullness	7 (29.2%)	0 (0.0%)
Tinnitus	8 (33.3%)	1 (4.2%)

## Discussion

Traumatic TMPs tend to heal spontaneously. However, some perforations result in poor tympanic membrane healing, which subsequently affects sound conduction. In the present study, pseudo-tympanic membrane healing occurred because the TMP was covered by thickened tissue and was thus mistaken for normal healing by head mirror visualization. Pseudo tympanic membrane healing differs from crust and atrophic healing. The tympanic membrane consists of an epithelial, fibrous and mucosal layer. The epithelial layer includes squamous epithelium, stratum corneum and a basement membrane. Two theories regarding the mechanism of spontaneous tympanic membrane healing have been proposed. In the first theory, the perforation is closed by keratin, which is produced by the epithelial layer in most of cases, followed by proliferation of the epithelial and fibrous layers.^13^, ^14^, ^15^, ^16^ The stratum corneum is the most important layer of the epidermis with respect to closure of the perforation; keratin products also play an important role in spontaneous healing.^13^ The perforation is first closed by connective tissue proliferation, and may subsequently be re-epithelialized.^17^, ^18^ Lou et al.^19^ believe that the healing tympanic membrane exhibits different patterns of granulation tissue formation and proliferating epithelium migration in dry and wet perforations; the healing begins with physiological migration of the proliferating epithelium under dry conditions, and with granulation tissue formation in wet environments. Lou^20^, ^21^ found that the healing of dry perforations causes outward epithelial cell migration and crust formation, which impedes perforation healing and prolongs the healing time. Poor healing of the tympanic membrane occurs mainly in dry perforations. Crust healing can be characterized by hyperplastic epithelium, desquamated keratin debris, and foreign body giant cells.^14^, ^22^ The tympanic membrane is less likely to completely heal in the absence of keratin and fibrous layers, i.e., when the perforation is only covered by thin and patchy crust tissue. An atrophic tympanic membrane is characterized by normal epithelial and keratin layers, with no fibrous layer. The healing mechanism of pseudo tympanic membrane healing is unclear.

Keratin products play a vital role in the healing process in dry perforations.^13^ We speculate that crust healing results from outward migration of hyperplastic epithelium, or by epithelium hyperactivity and decreased activity of the keratin layer. Johnson and Hawke^23^ believe that extensive disruption of the stratum corneum is less likely to facilitate TMP healing. However, pseudo-healing of the tympanic membrane can result from weak proliferation of the epithelial layer. Hyperactivity of the keratin layer resulted in excessive accumulation of keratin, thereby closing the perforation; however, normal epithelial and fibrous layers were not present. Alternatively, excessive epithelization of the keratin layer could have inhibited remodeling of the tympanic membrane during the healing process. Gladstone et al.^24^ believe that excessive epithelization of the keratin layer is not conducive to perforation healing. Boedts^25^ reported that excessive accumulation of keratin products disturbed epithelial migration due to mechanical pressure, and thereby impeded perforation closure. However, the exact mechanisms of pseudo-tympanic membrane healing require further study.

Eardrum healing involves the recovery of normal anatomical structures and proper sound conduction. The normal architecture and physical properties of tympanic membranes are of vital importance for hearing.^26^ Pseudo-healing of the tympanic membrane involves the loss of normal tympanic membrane morphology and increased tympanic membrane weight. This results in excess stiffness of the tympanic membrane, and reduced sound conduction and tympanic membrane vibration. In the present study, tympanometry identified A- and B-Type patients with pseudo-tympanic membrane healing, and pure-tone audiograms demonstrated normal or mild conductive hearing loss. However, all 24 pseudo-healing perforations in the present study achieved complete closure, with a mean closure time of 6.1 ± 2.3 days following EGF treatment. Kanemaru et al.^6^ treated 53 chronic TMPs using b-FGF and the closure rate achieved 98.1% (52/53). Previous experimental studies have suggested that EGF facilitates proliferation and migration of epithelial cells, as well as regeneration of blood capillaries. Different perforation margin phenotypes could influence the affinity of growth factors for different types of cell during the healing process. A previous study showed^27^ that FGF2 had a preference for the fibrous layer, thereby inducing the proliferation of fibroblasts and regulating the reaction of connective tissue during the proliferation stage; meanwhile, EGF stimulated the epithelial layer and promoted proliferation and migration of epithelial cells and keratinocytes during the proliferation stage. Thus, the characters of EGF address the defect of weaker epithelial regeneration and strong differentiation of stratum corneum for the perforation with pseudo healing. This promotes the pseudo healing to achieve normal healing. Ramsay et al.^12^ believe that EGF does not improve the closure of human chronic TMPs. They emphasize that the perforated edge must be removed to produce a fresh wound and thus improve the closure rate of chronic TMPs.^12^ In the present study, thickened tawny tissue was excised and a new perforated edge was formed before the EGF treatment, which is in accordance with the therapeutic principle of Ramsay et al.^12^ Destruction of microcirculation structures of the perforated edge and topical application of EGF may induce new inflammatory responses, which stimulate normal healing of the tympanic membrane and revascularization.^8^, ^22^ In addition, topical application of EGF can produce a moistened perforation edge and facilitate the proliferation of granulation tissue, thereby accelerating perforation healing.^17^, ^18^ Although surgery without use of the EGF application may produce the higher closure rate, surgery requires anesthesia and is an invasive treatment. We observed that the pseudo-healing of the perforations in this study was different from that of chronic perforations with Chronic Suppurative Otitis Media (CSOM). The presence of CSOM in chronic TMPs attenuates tympanic membrane healing, where recurrent inflammation of the middle ear and bacteriotoxins inhibits endogenous healing mechanisms, such that scar hyperplasia of dense fibrous connective tissue manifests at the perforation edge.^24^, ^28^ We believe that excision of thickened tawny tissue and topical application of EGF merely assisted and guided the normal tympanic membrane healing process. In addition, the toxicity of EGF has been investigated: previous studies showed that EGF caused no apparent risk of ototoxicity when topical application of EGF to the external and middle ears.^7^, ^9^, ^11^ Recent clinical studies demonstrated that neither sensorineural hearing loss nor external auditory canal stenosis resulted from short-term EGF application to tympanic membrane perforations.^8^, ^12^, ^27^, ^29^ Although animal studies demonstrated that continuous EGF application resulted in the development of middle ear cholesteatoma,^10^ in the present study, the dosage of application was only 0.1‒0.15 mL once daily to keep the tympanic membrane moist, the application time was 3–12 days, shorter than the application time of 6 weeks reported in a previous experimental study. No severe complications related to the use of EGF were observed during the treatment process in this study.

## Limitations

There were several limitations to the present study, such as the small sample size, the absence of saline-control group; moreover, histological examinations were not performed on the thickened tawny tissue covering the perforations. Future studies should be conducted to further characterize pseudo-healing of traumatic TMPs. In addition, it would be interesting to follow non-EGF treated patients to see if their outcomes improve over time, and compare those to treated patients.

## Conclusion

Although pseudo-healing of human traumatic TMPs is rare, it may affect sound conduction in the tympanic membrane, resulting in symptoms such as tinnitus, aural fullness, and ear discomfort. The topical application of EGF is a simple, convenient, low-cost (only approximately 3.7$) and noninvasive treatment, which could improve the closure rate of perforations showing pseudo-healing and thus achieve normal tympanic membrane morphology.

## Funding

This study was supported by the Science and Technology Agency of Zhejiang Province, China (Grants nº 2013C33176).

## Conflicts of interest

The author declares no conflicts of interest.
